# Mycothiol maintains the homeostasis and signalling of nitric oxide in *Streptomyces coelicolor* A3(2) M145

**DOI:** 10.1186/s12866-023-03036-z

**Published:** 2023-10-05

**Authors:** Tomoki Yoshizumi, Yukiko Shibui, Minori Kogo, Sota Honma, Shinsaku Ito, Shunsuke Yajima, Yasuyuki Sasaki

**Affiliations:** Department of Bioscience, Faculty of Life Sciences, Tokyo University of Agriculture, Sakuragaoka, Tokyo, Setagaya-Ku 156-8502 Japan

**Keywords:** Nitric oxide, Actinobacteria, Mycothiol, Intracellular homeostasis, Signal transduction, Secondary metabolism

## Abstract

**Background:**

Previous studies have revealed a nitric oxide (NO) metabolic cycle in which NO, nitrate (NO_3_^−^), and nitrite (NO_2_^−^) circulate. The NO produced in this cycle serves as a signalling molecule that regulates actinorhodin (ACT) production via the DevS/DevR NO-dependent two-component system (TCS) in *Streptomyces coelicolor* A3(2) M145. However, the mechanisms involved in the regulation of NO signalling in *S. coelicolor* have not yet been elucidated. Mycothiol (MSH), a thiol molecule produced by *Actinomyces*, is involved in the defence mechanisms against oxidative stress. Therefore, this study focused on the correlation between intracellular NO and MSH levels.

**Results:**

To investigate the interaction of MSH with endogenously produced NO, we generated an *S. coelicolor* A3(2) strain deficient in MSH biosynthesis. This mutant strain exhibited a decrease in low-molecular-weight S-nitrosothiols and intracellular NO levels during culture compared to those of the wild-type strain. Moreover, the mutant strain exhibited reduced activity of the DevS/DevR TCS, a regulator of NO homeostasis and ACT production, from the early stage of culture, along with a decrease in ACT production compared to those of the wild-type strain.

**Conclusions:**

This study suggests that MSH maintains intracellular NO homeostasis by forming S-nitrosomycothiol, which induces NO signalling. Finally, we propose a metabolic model in which MSH from endogenously produced NO facilitates the maintenance of both NO homeostasis and signalling in *S. coelicolor* A3(2) M145.

**Supplementary Information:**

The online version contains supplementary material available at 10.1186/s12866-023-03036-z.

## Background

Nitric oxide (NO) is a highly reactive and diffusible molecule that can easily permeate the cell membrane [1]. Since Palmer first reported its activity in 1987, NO has been recognised as a signalling molecule that regulates various physiological functions in most organisms [2]. NO mediates cell-to-cell communication and regulates several metabolic pathways by interacting with transition metals (e.g., Fe and Cu [3]), thiol groups [4], free-radical oxygen [5], and unsaturated fatty acids [6]. However, increased levels of NO exert cytotoxic effects owing to its high reactivity. NO reacts with O_2_^•−^ to form peroxynitrite (ONOO^−^), which leads to intracellular dysregulation due to the oxidation of a wide range of cellular molecules such as proteins, lipids, and nucleic acids [7]. Thus, cells must strictly regulate the internal NO concentration to ensure their viability.

Several studies have reported mechanisms involved in the regulation of intracellular NO levels. NO production by enzymes such as nitric oxide synthase and nitrite reductase, which reduce nitrite to NO, depends on the transient expression of genes [8, 9]. To protect cells from NO toxicity, flavohaemoglobin (Fhb) and nitric oxide reductase eliminate cellular NO by converting it into nitrate and nitrous oxide, respectively [10, 11]. Additionally, low-molecular-weight (LMW) thiol compounds such as glutathione (GSH) and thionein modulate the NO concentration by generating S-nitrosothiol [12, 13]. These small molecules can therefore protect cells against nitrosylative stress and regulate the S-nitrosylation of proteins and their corresponding functions.

*Streptomyces* species are gram-positive soil-dwelling filamentous bacteria that exhibit a complex life cycle similar to that of fungi. These bacteria produce many commercially available secondary metabolites, including anticancer agents [14], agrochemicals [15], and antibiotics [16]. Recently, we reported an NO metabolic cycle in which NO, nitrate, and nitrite are circulated by Fhb and nitrate reductase (Nar) in *Streptomyces coelicolor* A3(2) M145; the NO produced in this cycle serves as a signalling molecule to regulate morphological differentiation and secondary metabolism in bacteria [17]. In addition, the production of NO and actinorhodin (ACT; a blue-pigmented antibiotic) were demonstrated to be regulated by the NO-dependent two-component regulatory system (TCS) DevS/DevR in *Streptomyces coelicolor* A3(2) M145 [18]. The sensor protein DevS binds NO and phosphorylates DevR, a cognate response regulator. Phosphorylated DevR then activates the expression of its own genes (*devR*, *narG2*, and *actII-ORF4*). In addition, DevS senses NO elevation during cell growth and inactivates its autophosphorylation activity, thereby inhibiting the NO synthesis pathway. Thus, DevS/R functions as an autoregulatory system to maintain NO homeostasis in *S. coelicolor* A3(2) M145 [18].

Mycothiol (MSH) is an *Actinomycetes*-specific LMW thiol compound consisting of an acetylated cysteine linked to glucosamine and inositol [19]. Additionally, MSH acts as an antioxidant buffer by forming mycothione, which is reduced by disulphide reductase [20]. Similar to GSH, MSH reacts with NO to form S-nitrosomycothiol (MSNO) and mitigates reactive nitrogen species-induced damage [21]. Four enzymes [MshA (SCO4204), MshB (SCO5126), MshC (SCO1663), and MshD (SCO4151)] are involved in the MSH biosynthesis pathway in *S. coelicolor* A3(2) M145 [22]. Although the correlation between LMW thiol compounds and oxidative stress has been extensively studied, the relationship between MSH and intracellular NO signalling in *S. coelicolor* A3(2) M145 remains unclear. In the present study, we investigated the relationship between MSH and endogenously produced NO in *S. coelicolor* A3(2) M145 cells.

## Results

### Effect of MSH deficiency on cell growth

**Fig. 1:**
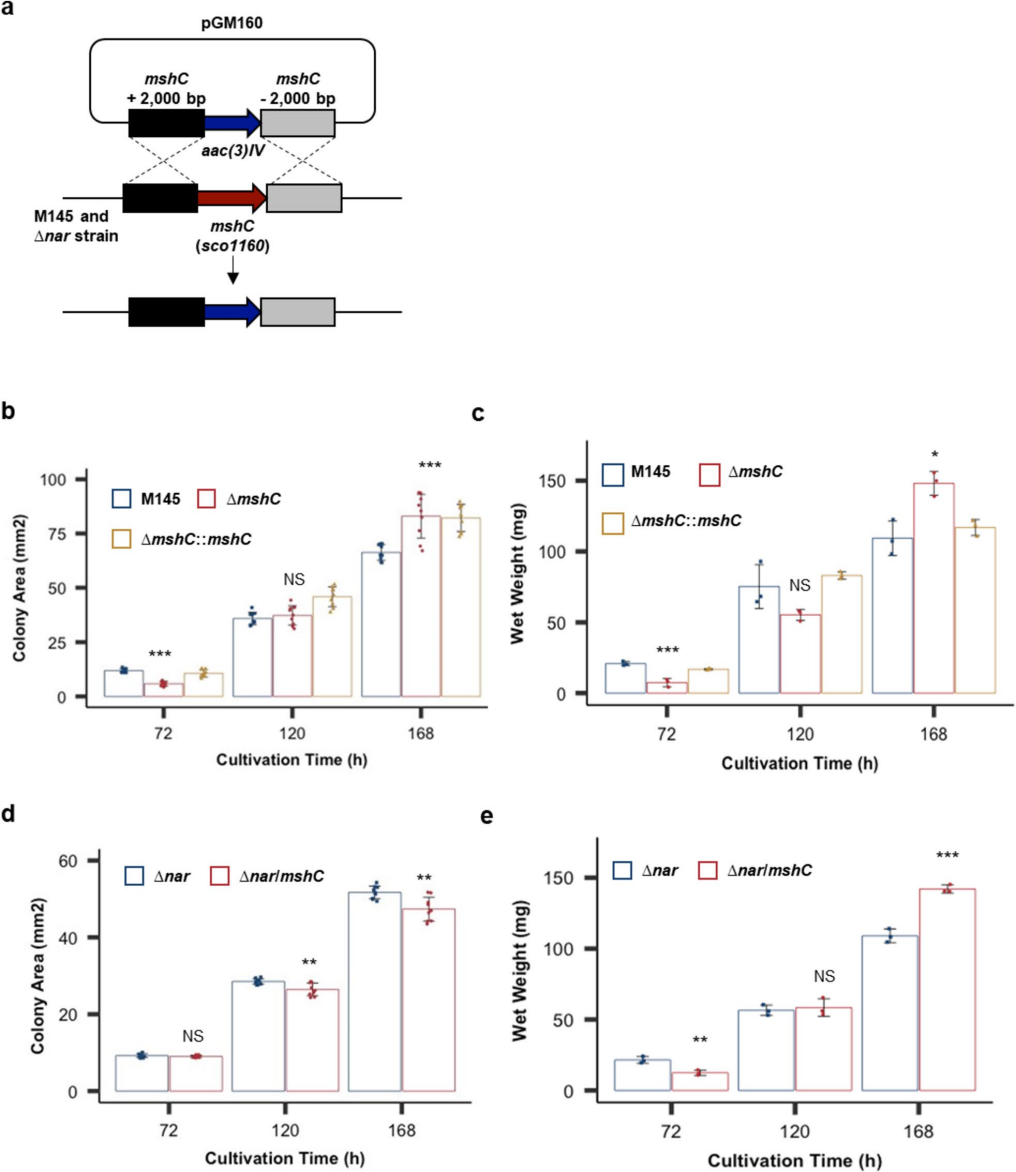
Effect of mycothiol (MSH) deficiency on colony growth. **a** Construction of the *mshC* disruptant strain. The plasmid pGM160, including the 2,000 nucleotides upstream and downstream of *mshC* flanking an *aac(3)IV* gene, was transformed into *Streptomyces coelicolor* A3(2) M145. The double-crossover event is shown schematically. **b** The colony area of strains M145 (blue bars), *∆mshC* (red bars), and *∆mshC::mshC* (yellow bars) cultured for the indicated durations. Data are presented as mean and standard deviation (*n* = 9). ***p < 0.001; NS, not significant (Student’s t-test). **c** The wet weight of M145 (blue bars), *∆mshC* (red bars), and *∆mshC::mshC* (yellow bars) strains cultured for the indicated durations. Data are presented as mean and standard deviation values from three independent experiments. ***p < 0.001; *p < 0.05. NS, not significant (Student’s t-test). **d** The colony area of *∆nar* (blue bars) and *∆nar/mshC* (red bars) strains cultured for the indicated durations. Data are presented as mean and standard deviation (*n* = 9) from three independent experiments. **e** The wet weight of *∆nar* (blue bars) and *∆nar/mshC* (red bars) strains cultured for the indicated durations. Data are presented as the mean and standard deviation values from three independent experiments. ***p < 0.001; **p < 0.01; NS, not significant (Student’s t-test)

Previous studies have reported that MSH eliminates reactive oxygen and nitrogen species generated in cells [20, 21]. However, the relationship between MSH expression and endogenous NO production remains unclear. A *Streptomyces coelicolor* A3(2) M145 strain lacking MSH synthesis-related genes (*mshA*, *mshC*, and *mshD*) cannot synthesize MSH [22]. Thus, we deleted *mshC* by homologous recombination using the plasmid pGM160 (Fig. 1a) to obtain a model strain incapable of MSH biosynthesis.

To evaluate the effect of the loss of MSH biosynthesis ability in the cells, we comparatively investigated the colony areas and wet weights of the wild-type (WT) M145 strain and MSH-deficient ∆*mshC* strain cultured on yeast extract-malt extract-glutamine (YEME-gln) agar medium using ImageJ. Only relatively small differences in colony area and wet weight were found between strains after 72 and 168 h of incubation, respectively (Fig. 1b, c). Furthermore, only slight differences in colony area and wet weight were also found between the *nar* deletion strain (∆*nar* strain), with reduced NO production ability, and the *nar* and *mshC* double-deletion strains (∆*nar*/*mshC* strain) (Fig. 1d, e). These results suggested that MSH has a relatively small effect on the growth of *S. coelicolor* A3(2) M145, regardless of the presence or absence of endogenously produced NO.

### Deficiency of MSH synthesis decreases the intracellular NO level in *S. coelicolor* A3(2)M145

**Fig. 2:**
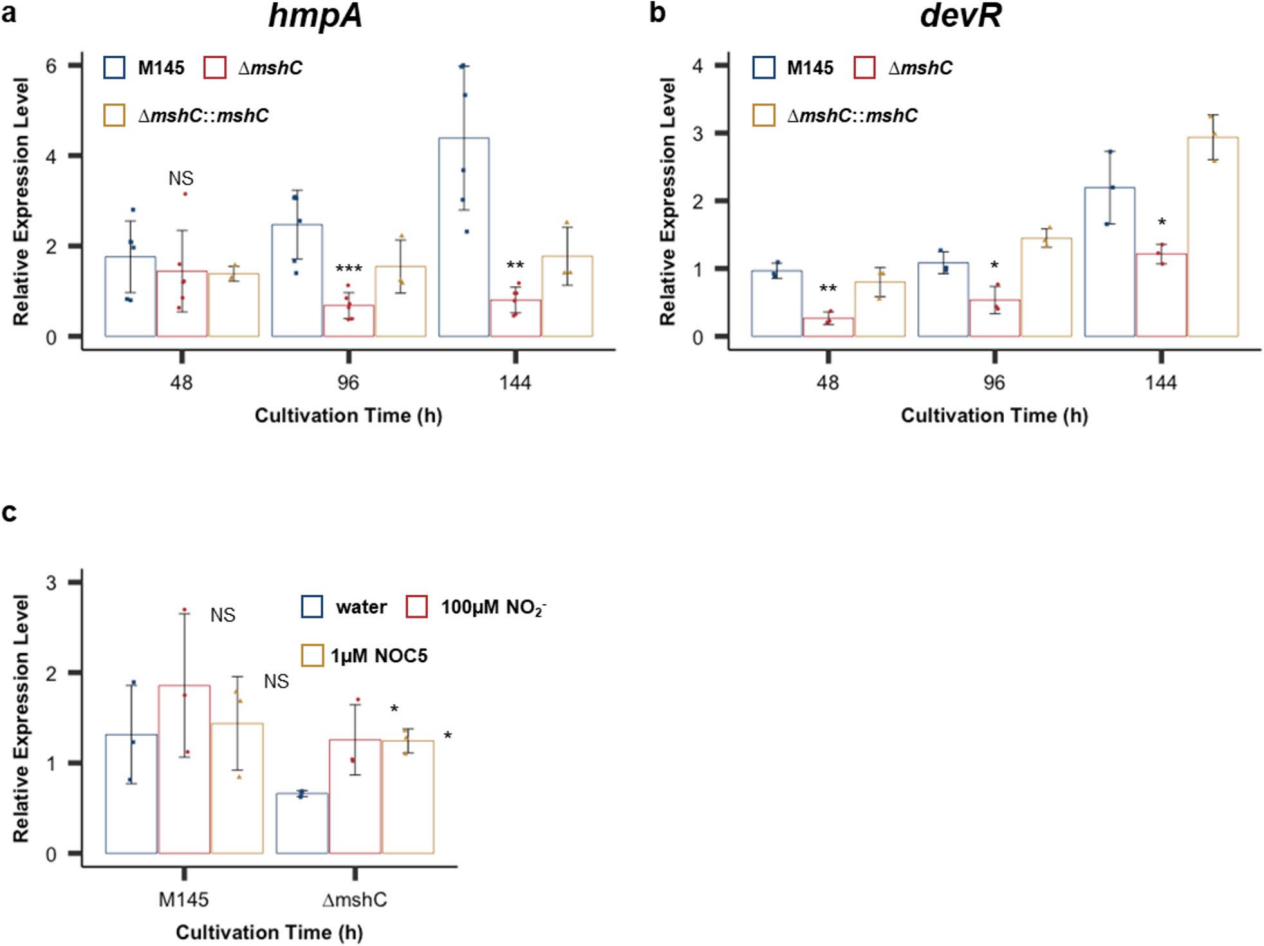
Mycothiol (MSH) gene deletion decreases intracellular nitric oxide (NO) levels. **a** Reverse-transcription quantitative polymerase chain reaction (RT-qPCR) analysis of the relative expression levels of *hmpA* in the total RNA extracted from strains M145 (blue bars), ∆*mshC* (red bars), and ∆*mshC*::*mshC* (yellow bars) cultured for the indicated durations. Data are presented as the mean and standard deviation values from six independent experiments. **p < 0.01; *p < 0.05; NS, not significant (Student’s *t*-test). **b** Relative mRNA expression levels of *devR* in the total RNA extracted from strains M145 (blue bars), ∆*mshC* (red bars), and ∆*mshC*::*mshC* (yellow bars) cultured for the indicated durations. Data are presented as the mean and standard deviation values from three independent experiments. **p < 0.01, *p < 0.05 (Student’s *t*-test). **c** Relative mRNA expression levels of *hmpA* in the total RNA extracted from M145 and *∆mshC* strains. After the strains were cultured for 72 h, they were treated with 1 ml water (control; blue bars), 100 μM NO_2_.^−^ for 1 h (red bars), or 1 μM NOC5 solution (yellow bars); incubated for 30 min; and subjected to RT-qPCR analysis. Data are presented as mean and standard deviation values from three independent experiments. *p < 0.05; NS, not significant (Dunnett’s test)

Because *hmpA* (encoding Fhb) is regulated by the NO-dependent transcription factor NsrR, the gene expression level is reflective of the intracellular NO concentration [23]. To verify the effect of MSH synthesis deficiency on endogenous NO levels, we examined the expression levels of *hmpA* in the M145 and ∆*mshC* strains. The transcription levels of *hmpA* increased in a time-dependent manner in both strains but were maintained at lower levels in the ∆*mshC* strain compared to those in the M145 strain (Fig. 2a).

DevS/R has been reported to regulate NO synthesis in a dose-dependent manner to maintain NO homeostasis [18]. Therefore, we further examined NO homeostasis in the ∆*mshC* strain by evaluating the expression of the DevS/R TCS regulon *devR*. The transcription level of *devR* in the ∆*mshC* strain was decreased compared to that in the M145 strain during culture (Fig. 2b). These results suggest that the lack of MSH suppressed NO production in the ∆*mshC* strain during the early stages of culture. This was supported by two exogenous NO donors inducing the expression of *hmpA* in the ∆*mshC* strain (Fig. 2c).

### MSH deletion decreases LMW S-nitrosothiol levels

**Fig. 3:**
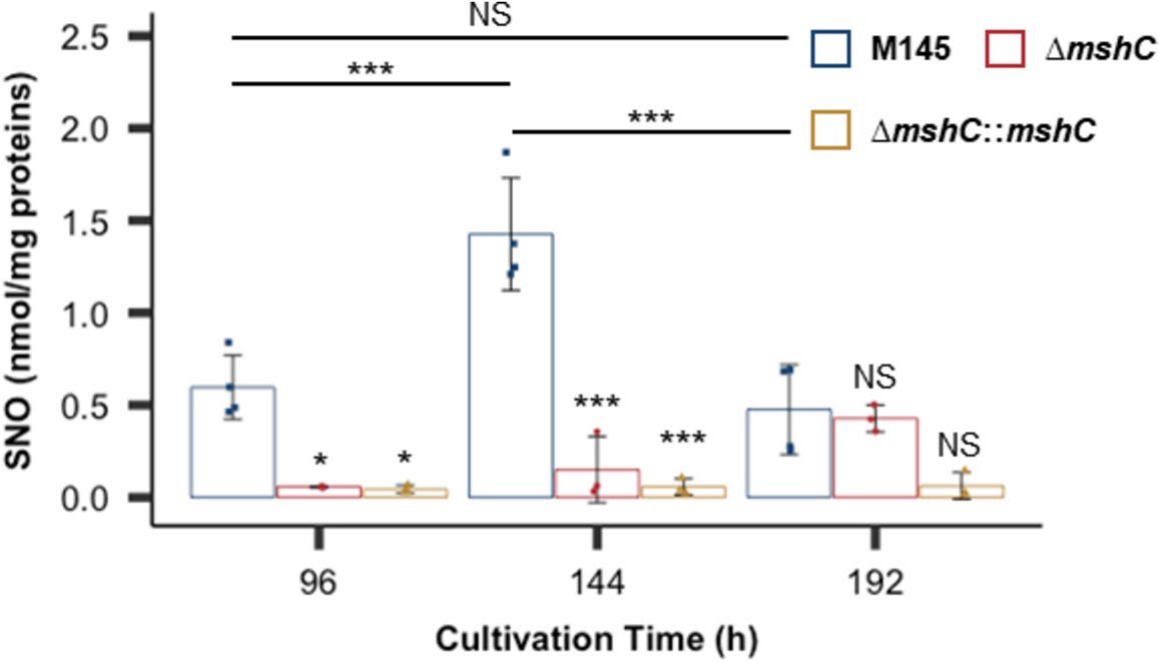
Defect of mycothiol (MSH) synthesis decreases low-molecular-weight (LMW) S-nitrosothiol (SNO) levels. M145 (blue bars), ∆*mshC* (red bars), and ∆*nar* (yellow bars) strains were cultured for the indicated durations. After deproteinisation, SNO was converted to NO_2_^−^ using Hg.^2+^ as a catalyst. SNO levels were measured using the Saville–Griess assay. Concentrations below the detection limit (500 nM) were recorded as the detection limit concentration. Data are presented as the mean and standard deviation values from three or four independent experiments. ***p < 0.001; *p < 0.05; NS, not significant (Tukey–Kramer test)

Since previous studies demonstrated that S-nitrosothiols are formed by thiol groups that bind to NO in the presence of electron acceptors in cells [24, 25], we verified LMW S-nitrosothiol levels in the M145, ∆*mshC*, and ∆*nar* strains in a time-dependent manner. The LMW S-nitrosothiol levels increased from 96 to 144 h but decreased after 144 h of culture in the M145 strain (Fig. 3). This is consistent with the results of a previous study demonstrating time-dependent changes in NO production in vivo [18]. Intracellular NO levels in *S. coelicolor* A3(2) M145 increased during the middle phase of culture and decreased during the late phase. In contrast, the LMW S-nitrosothiol levels in the ∆*mshC* and ∆*nar* strains were lower than those in the M145 strain between 96 and 144 h (Fig. 3). These results indicated that the amount of LMW S-nitrosothiols is positively correlated with intracellular NO levels in *S. coelicolor* A3(2) M145.

### MSH is needed for optimal ACT synthesis

**Fig. 4:**
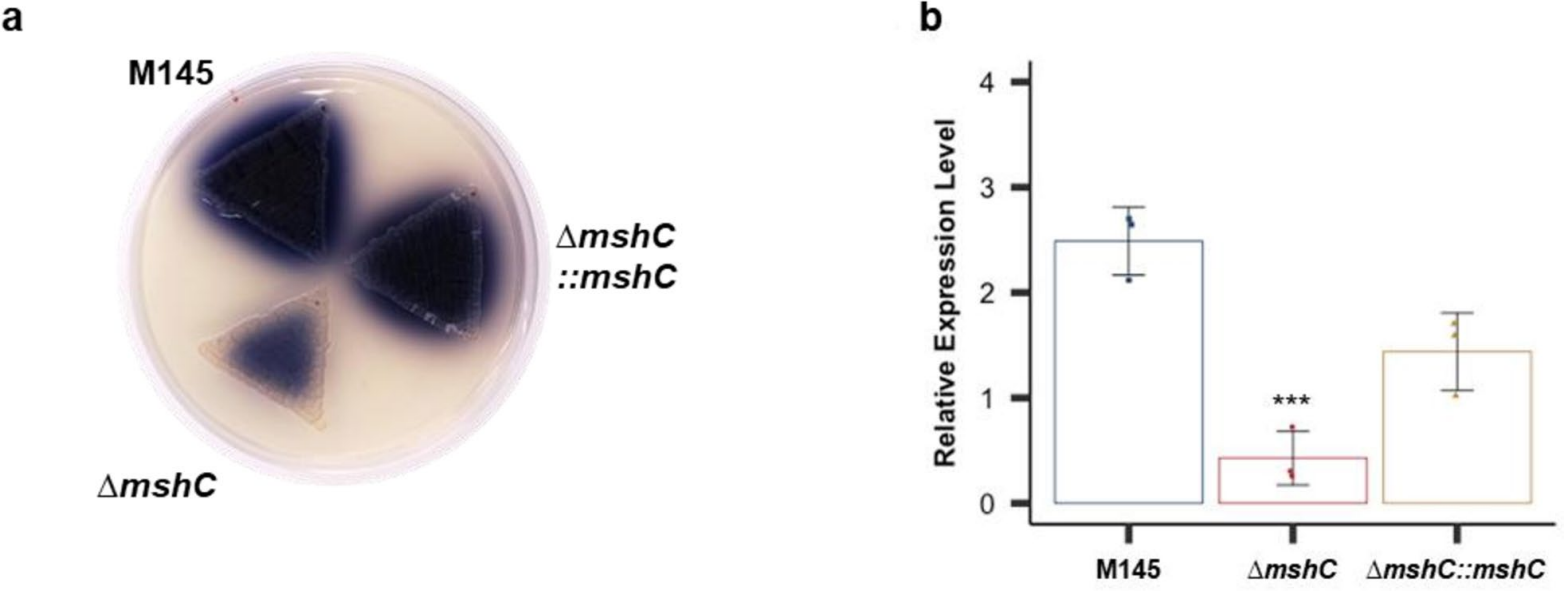
Mycothiol (MSH) is needed for optimal actinorhodin (ACT) synthesis. **a** Spores were inoculated onto yeast extract-malt extract-glutamine agar medium with a toothpick and cultured for 96 h at 30 °C. **b** M145 (blue bar), ∆*mshC* (red bar), and ∆*mshC*::*mshC* (yellow bar) strains were cultured under the same conditions as those in (a). Total RNA was extracted from each strain to measure the relative expression level of *actII-ORF4* using reverse transcription-quantitative polymerase chain reaction. Data are presented as mean and standard deviation values from three independent experiments. ***p < 0.001 (Student’s *t*-test)

A previous study demonstrated that the DevS/R TCS modulated ACT synthesis by regulating *actII-ORF4* expression levels [18]. Since we found that the lack of MSH affected cellular NO levels, ACT production and *actII-ORF4* expression levels were examined in the M145 and ∆*mshC* strains. Minimal ACT production (Fig. 4a) and downregulated *actII-ORF4* expression (Fig. 4b) were found in the ∆*mshC* strain inoculated on YEME-gln agar and cultured for 96 h. These results suggest that the lack of MSH negatively affects NO signalling and ACT production in *S. coelicolor* A3(2) M145 cells.

Thus, MSH is essential for maintaining intracellular NO homeostasis and for mediating NO signalling in *S. coelicolor* A3(2) M145.

## Discussion

As NO is highly reactive, its cellular concentration must be strictly regulated to exert its physiological functions. The results of the present study indicate that MSH-mediated intracellular NO homeostasis facilitates optimal NO signalling in bacteria. The GSH synthesis pathway has not been detected in *Actinomycetes*; however, members of this bacterial genus produce MSH as their major redox buffer and coenzyme [26–28]. MSH-deficient mutants of *Mycobacterium smegmatis* exhibit increased sensitivity to exogenous NO [29], reactive oxygen species [30], alkali agents, and antibiotics [31]. *Mycobacterium tuberculosis* relies on MSH-dependent systems to infect macrophages [32]. *Corynebacterium glutamicum* metabolises aromatic compounds that require MSH as a coenzyme [33]. Thus, *Actinomycetes* use MSH to adapt to their environments. In addition, an MSH-defective strain of *S. coelicolor* A3(2) was reported to exhibit high sensitivity to hydrogen peroxide [34].

While being statistically significant, the differences in cell growth between the M145 and ∆*mshC* strains remained relatively small during culture. However, the lack of MSH due to *mshC* deletion disrupted intracellular NO homeostasis. These results suggest that MSH acts not only as a defence molecule against reactive oxygen species but also as a mediator involved in maintaining intracellular NO homeostasis.

Although previous studies have revealed that bacteria endogenously produce NO and possess regulatory mechanisms for NO-mediated metabolism, these are transient adaptive responses to environmental changes [35–38]. *Streptomyces coelicolor* A3(2) M145 scavenges endogenously produced NO via MSH, in addition to Fhb-mediated NO metabolism. NO signalling can start gradually as intracellular NO levels overcome the metabolisable levels of both mechanisms. Therefore, *S. coelicolor* A3(2) M145 possesses an advanced mechanism for regulating intracellular NO homeostasis.

We further found that the ACT production level in the ∆*mshC* strain was lower than that in the WT strain. The DevS/R activity in the ∆*mshC* strain was also lower than that in the WT strain. ACT production is regulated by NO signalling via the DevS/R TCS [18]. Therefore, the dysregulation of ACT production in the ∆*mshC* strain can be explained by the upregulation of intracellular NO-induced DevS/R repression in the absence of MSH.

Thiols bind directly to NO under aerobic conditions [39] or in the presence of electron acceptors such as NAD^+^, metals, and metalloproteins in vivo [24, 25]. GSH forms S-nitrosoglutathione (GSNO) with the intracellular NO produced by nitric oxide synthase to maintain the life cycle of mammalian cells [40]. GSNO is a stable NO-carrying molecule in both plant and animal cells that regulates protein activity by transferring an NO group to cysteines [41–43]. LMW S-nitrosothiols function as donors of NO and nitrosonium ions and are involved in cellular signalling [44, 45]. S-Nitrosothiols are required for cell survival. In particular, GSNO interacts with specific thiol groups of proteins to produce high-molecular-weight S-nitrosothiols in a process called S-transnitrosation [46] and regulates the secondary metabolite content through protein S-nitrosylation [47].

LMW S-nitrosothiols were detected in the WT M145 strain. In contrast, LMW S-nitrosothiols were almost undetectable in the ∆*mshC* strain. This suggests that MSH interacts with endogenously produced NO and regulates the intracellular NO concentration through MSNO formation; however, we did not identify MSNO in the present study. In the future, it will be important to elucidate the mechanisms by which LMW S-nitrosothiols, including MSH, affect NO metabolism in actinomycetes.

The LMW S-nitrosothiol levels were not significantly different between the M145 and ∆*mshC* strains at 192 h, suggesting that the amount of other LMW thiols increased instead of MSH and that these thiols were involved in maintaining intracellular NO levels in the late stages of culture. This is consistent with the results of a previous study, which demonstrated that the amounts of other thiol compounds increased in an MSH-deficient strain of *S. coelicolor* A3(2) [34]. However, the levels of LMW S-nitrosothiols in the *mshC* strain were lower than those in the M145 strain at both 96 and 144 h. MSH deficiency inactivated the DevS/R TCS from the early stages, indicating that MSH is the main LMW thiol for maintaining NO homeostasis in the early stages of culture, which is necessary for signalling secondary metabolism in *S. coelicolor* A3(2) M145. *Actinomycetes* synthesise ergothioneine, an LMW thiol produced by Actinobacteria, in addition to MSH [48]. Thus, to understand the homeostatic regulation of NO in *Actinomycetes*, the roles of other thiols in NO homeostasis must be considered.

This study suggests that intracellular NO homeostasis, which must be strictly maintained for optimal secondary metabolism, depends on MSH, a novel component that regulates NO signalling. MSH deficiency decreased ACT production. Elucidation of the regulatory mechanism of secondary metabolism will contribute to the efficient improvement of fermentation products. Additionally, *M. tuberculosis* has a similar form of NO metabolism [49, 50]. Therefore, identifying the role of MSH in maintaining intracellular NO homeostasis will enable the development of clinical therapeutic strategies against *M. tuberculosis* infections. Future studies should focus on the regulation of NO homeostasis through various mechanisms in several organisms, which can provide novel perspectives on NO biology and improvement of human health.

## Conclusions

**Fig. 5:**
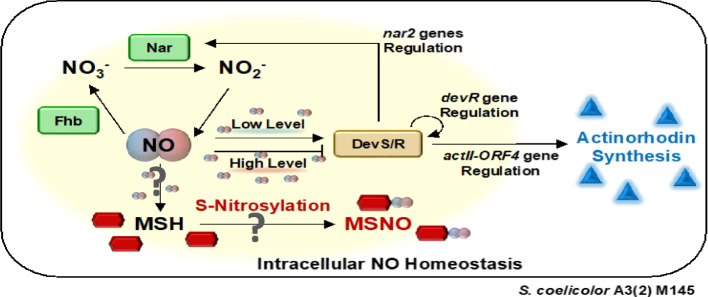
Proposed model of intracellular nitric oxide (NO) homeostasis in *Streptomyces coelicolor* A3(2) M145. Flavohaemoglobin (Fhb) and nitrate reductase (Nar) mediate the circulation of NO, NO_3_^−^, and NO_2_.^−^. DevS/R is activated or inactivated depending on intracellular NO levels. NO directly regulates actinorhodin synthesis through the DevS/R two-component regulatory system, which modulates *actII-ORF4* expression. DevR also regulates *nar2* expression and NO production. Intracellular NO homeostasis is maintained through Fhb metabolism and its interaction with mycothiol (MSH) (S-nitrosylation) to form S-nitrosomycothiol (MSNO)

This study suggests that MSH regulates intracellular NO homeostasis by promoting MSNO formation. MSH-mediated maintenance of NO homeostasis, which is dependent on intracellular NO levels, is important for ACT production in *S. coelicolor* A3(2) M145 under NO-producing conditions (Fig. 5).

## Methods

### Bacterial strains, plasmids, and culture conditions

The *S. coelicolor* A3(2) M145 parental strain and all mutants used in this study are listed in Table 1. To induce sporulation, the strains were cultured on mannitol soya flour agar (2% mannitol, 2% soya flour, and 2% agar) at 30 °C. Additionally, the strains were incubated for each experiment in YEME-gln agar medium (0.3% yeast extract, 0.3% malt extract, 0.5% bacto-peptone, 1% glucose, 50 mM l-glutamine, and 2% agar, pH 7.2) at 30 °C. As NO easily diffuses and affects strains grown in the same incubator, the ∆*nar* and ∆*nar/mshC* strains were incubated independently. Detailed culture conditions are described in the figure legends. *Escherichia coli* DH5α and *E. coli* HST04 *dam*^*−*^/*dcm*^*−*^ (TaKaRa Bio) strains were used as hosts for cloning and cultured in Luria–Bertani broth (1% tryptone, 0.5% yeast extract, and 0.5% NaCl) at 37 °C.

**Table 1:** Strains used in this study

*Streptomyces coelicolor* A3(2) M145 strain	Genotype and/or characteristics	Reference or source
M145 (wild type)	Lacks native plasmids, SCP1 and SCP2	[17]
∆*nar*	M145 SCO6535::*hyg*/SCO0216::*scar*/SCO4947::*scar* (*narGG2G3* removed)	[17]
∆*mshC*	M145 SCO1663::*aac(3)IV* (*mshC* removed)	This study
∆*mshC*::*mshC*	M145 SCO1663::*aac(3)IV*/pku460-SCO1163 (complementation of *mshC*)	This study
∆*nar*/*mshC*	M145 SCO6535::*hyg*/SCO0216::*scar*/SCO4947::*scar*/SCO1663::*aac(3)IV* (*narGG2G3* and *mshC* removed)	This study

### Construction of the ∆*mshC* and complementation strains

Two fragments corresponding to the upstream and downstream regions of *mshC* (*sco1663*) and a fragment corresponding to the apramycin-resistance cassette [51] flanked by flippase recognition target sites were polymerase chain reaction (PCR)-amplified with PrimeSTAR GXL DNA polymerase (TaKaRa Bio) using specific primer sets (Table 2), along with the introduction of *Eco*RI and *Hind*III restriction sites. The two fragments were ligated into the *Sma*I-digested pUC19 plasmid using Ligation High Ver. 2 (TOYOBO). The two products were transformed into *E. coli* DH5α competent cells (TaKaRa Bio). The plasmids were purified from *E. coli* cells, digested with *Eco*RI and *Hind*III, and the two fragments were ligated into the *Eco*R I-digested pKU450 plasmid using Ligation High Ver. 2. The resulting plasmids were transformed into *E. coli* DH5α competent cells. The plasmids were then purified. After digestion of the apramycin-resistance cassettes and plasmids with *Hind*III, the two fragments were ligated using Ligation High Ver. 2. The resulting products were transformed into *E. coli* DH5α competent cells. The cosmids were purified, digested with *Eco*RI, and ligated with *Eco*RI-digested pGM160 using Ligation High Ver. 2. The resulting construct was introduced into *E. coli* HST04 *dam*^*−*^/*dcm*^*−*^ competent cells (TaKaRa Bio) to obtain non-methylated cosmids. Purified cosmids were introduced into the *S. coelicolor* A3(2) M145 parental strain or each mutant. The drug-resistant recombinant strains were screened. Genomic DNA was checked for DNA recombination by PCR with the appropriate primer sets (Table 2).

**Table 2:** Primers used in this study

Primer name	Sequence (5′ → 3′)	Purpose
hrdB F	GCATGCTCTTCCTGGACCTCAT	qPCR
hrdB R	TGGAGAACTTGTAGCCCTTGGTGTA	qPCR
hmpA F	AACAGTCCGTTCCCGTGGT	qPCR
hmpA R	CGAAGAGCTTGCGGTAGAACAG	qPCR
actII-ORF4 F	AAAGGAATATCGCGCACCTGGAAG	qPCR
actII-ORF4 R	GTTCCGGAATCATCGGCCCTATTC	qPCR
devR F	ACGACGAACCGGACATCACC	qPCR
devR R	CGTCGTCGAAGGAGGTCAGC	qPCR
mshA F	GTGAGCCAGTACGTCAGCAG	qPCR
mshA R	AGCTCCACGATGTAGACGTTC	qPCR
mshB F	TCGCGAAGGTCTACTGGAAC	qPCR
mshB R	CCTTCTCGAACGGCAGTCC	qPCR
mshC F	CGTACGACGCCACCCACAT	qPCR
mshC R	TAGTGGACCTGCCGCTTGGT	qPCR
mshD F	TTGACCGGCCTGGACCTG	qPCR
mshD R	TGAGCGACCCCTGTTCGG	qPCR
∆mshC upstream F	AAGCTTTAAACAGGCGTACAGACAGAAGAAAGA	Disruption
∆mshC upstream R	GAATTCACCCCGATCGACAAGTACCG	Disruption
∆mshC downstream F	GAATTCGCCGAACTGGGGAACGAG	Disruption
∆mshC downstream R	AAGCTTCATGTCATGAGCCTAACCGGA	Disruption
FRT drug resi F	CTCGAGAAGCTTATTCCGGGGATCCGTCGACC	Disruption
FRT drug resi R	CTCGAGAAGCTTTGTAGGCTGGAGCTGCTTC	Disruption
comp mshC F	CTCGAGGAATTCCGCTGAAGGACGGCGGCA	Complementation
comp mshC R	CTCGAGAAGCTTTTACAGCGCCACGCCCAGC	Complementation

*qPCR* quantitative polymerase chain reaction

The pKU460 plasmid was used for genetic complementation of the knockout mutants. The complementary cassette was obtained by PCR using the complementary primer set (Table 2) and ligated to the *Sma*I-digested pUC19 plasmid using Ligation High Ver. 2. The ligation product was introduced into *E. coli* DH5α competent cells, and the cosmid pUC19-*mshC* was purified and ligated with pKU460. The ligation product pKU460-*mshC* was introduced into *E. coli* HST04 *dam*^*−*^/*dcm*^*−*^ competent cells to obtain a non-methylated cosmid. The obtained cosmid was then introduced into the ∆*mshC* strain. The complementary mutant was verified by PCR using the appropriate primer sets (Table 2) and a complementation study.

### Measurement of colony areas

Spores were incubated on the YEME-gln agar medium at intervals of 1.8 cm (total of nine spots, 3 × 3 squares) using a sterilised toothpick. After incubating at 30 °C, a line of colonies (three colonies) including the central colony were photographed under a microscope (OLYMPUS) at a 7 × multiplier, and then the nine colony areas for three plates were measured using ImageJ (Version 1.53t, 24 August 2022 upgrade).

### Measurement of cell wet weight

Spores were incubated on YEME-gln agar medium at intervals of 1.8 cm (total of nine spots, 3 × 3 squares) using a sterilised toothpick. After incubating at 30 °C, all colonies were collected in a microtube while carefully removing the agar medium with a toothpick and then the cell wet weight was measured using a precision balance.

### S-nitrosothiol quantification

Spores were incubated on YEME-gln agar medium at intervals of 1.8 cm (total of nine spots, 3 × 3 squares) using a sterilised toothpick. After incubating at 30 °C, all colonies were collected in a 10 ml conical tube while carefully removing the agar medium with a toothpick.

S-nitrosothiol was measured using the Griess assay [52]. Cells were lysed in extraction buffer (0.1 M Tris–HCl, pH 7.2) on ice using sonication. The lysate was centrifuged at 20,000 × *g* and 4 °C for 15 min. Protein concentrations were measured using Bradford Protein assay kit (BIO-RAD) [53]. To precipitate proteins, the cell-free extract was incubated with acetone at − 20 °C for 15 min. The samples were then centrifuged at 20,000 × *g* and 4 °C for 10 min. Acetone was removed using an evaporator. The sample was incubated with an equivalent volume of buffer A (0.5 M HCl, 1% sulphanilamide) or buffer B (0.5 M HCl, 1% sulphanilamide, 0.2% HgCl_2_) for 15 min at 20–25 °C, followed by incubation with an equal volume of Griess reagent [0.5 M HCl, 0.02% *N*-(1-naphtyl)-ethylenediamine dihydrochloride] for 15 min. S-nitrosothiol was quantified by measuring the absorbance of the reaction mixture at 540 nm. A standard curve was constructed using different concentrations (0, 1, 5, 10, 15, and 20 μM) of GSNO in 50 μM NaNO_2_. Concentrations below the detection limit (500 nM) [54].

### RNA extraction and reverse transcription-quantitative PCR (RT-qPCR)

Spores were inoculated on YEME-gln agar medium at intervals of 1.8 cm (total of nine spots, 3 × 3 squares) using a sterilised toothpick. After incubating at 30 °C, all colonies were collected in a microtube while carefully removing the agar medium with a toothpick. Total RNA was purified from cells using the ReliaPrep RNA Cell Miniprep System (Promega). The isolated RNA was reverse-transcribed into complementary DNA using ReverTra Ace RT-qPCR Master Mix with gDNA Remover (TOYOBO), which was used as a template for qPCR analysis. The primers used for RT-qPCR analysis are listed in Table 2. qPCR was performed using the Thermal Cycler Dice Real-Time System (TaKaRa Bio). The PCR mixture (20 μL) contained 1 μg cDNA, 10 pmol of an appropriate primer set (Table 2), and THUNDERBIRD SYBR qPCR Mix (TOYOBO) or KOD SYBR qPCR Mix (TOYOBO). The expression levels of the target genes were normalised to those of *hrdB*, a housekeeping gene.

### ACT production and RNA extraction

The strains were inoculated in a triangular shape in the YEME-GLN medium. The ACT-derived blue pigment was visualised using a camera (Canon). Total RNA was extracted from the cells using the ReliaPrep RNA Cell Miniprep System (Promega) by the same technique as described above.

### Statistical analysis and reproducibility

All bars in figures represent the mean values obtained from at least three independent biological experiments and error bars represent the standard deviation. The detailed sample number (*n*) is provided in the legend of each figure. Differences in means between strains or conditions were analysed using Student’s* t*-test, Dunnett’s test, or the Tukey–Kramer test, depending on the comparison items. Differences were considered statistically significant at *p* < 0.05.

### Supplementary Information

**Additional file 1.**

## Data Availability

The dataset supporting the conclusions of this study is included in the article and an additional file (Additional file 1). All data will be made available from the corresponding author upon request.

## Abbreviations

ACT: Actinorhodin
Fhb: Flavohaemoglobin
GSH: Glutathione
GSNO: S-nitrosoglutathione
LMW: Low molecular weight
MSH: Mycothiol
MSNO: S-nitrosomycothiol
Nar: Nitrate reductase
NO: Nitric oxide
PCR: Polymerase chain reaction
RT-qPCR: Reverse transcription-quantitative polymerase chain reaction
TCS: Two-component system
WT: Wild type
YEME-gln: Yeast extract-malt extract-glutamine
